# Containment of SARS-CoV-2 Delta strain in Guangzhou, China by quarantine and social distancing: a modelling study

**DOI:** 10.1038/s41598-022-21674-7

**Published:** 2022-12-06

**Authors:** Zhiqi Zeng, Tong Wu, Zhijie Lin, Lei Luo, Zhengshi Lin, Wenda Guan, Jingyi Liang, Minfei Yu, Peikun Guan, Wei He, Zige Liu, Guibin Lu, Peifang Xie, Canxiong Chen, Eric H. Y. Lau, Zifeng Yang, Chitin Hon, Jianxing He

**Affiliations:** 1 State Key Laboratory of Respiratory Disease, National Clinical Research Center for Respiratory Disease, Guangzhou Institute of Respiratory Health, The First Affiliated Hospital of Guangzhou Medical University, Guangzhou, Guangdong 510120 People’s Republic of China; 2 Guangzhou key laboratory for clinical rapid diagnosis and early warning of infectious diseases, KingMed School of Laboratory Medicine, Guangzhou Medical University, Guangzhou, People’s Republic of China; 3 Macao Institute of Systems Engineering, Macao University of Science and Technology, Macau SAR, People’s Republic of China; 4 Singou Technology (Macau) Ltd, Macau SAR, People’s Republic of China; 5 Guangzhou Center for Disease Control and Prevention, Guangzhou, Guangdong People’s Republic of China; 6 Faculty of Life Science and Technology, Kunming University of Science and Technology, Kunming, Yunnan 650500 People’s Republic of China; 7 School of Public Health, Li Ka Shing, Faculty of Medicine, The University of Hong Kong, 7 Sassoon Road, Pokfulam, Hong Kong People’s Republic of China; 8 Laboratory of Data Discovery for Health, Tai Po, Hong Kong People’s Republic of China; 9 Guangzhou Laboratory, Guangzhou, People’s Republic of China

**Keywords:** Computational models, Epidemiology

## Abstract

China detected the first case of severe acute respiratory syndrome coronavirus 2 (SARS-CoV-2) infection with Delta variant in May 2021. We assessed control strategies against this variant of concern. We constructed a robust transmission model to assess the effectiveness of interventions against the Delta variant in Guangzhou with initial quarantine/isolation, followed by social distancing. We also assessed the effectiveness of alternative strategies and that against potentially more infectious variants. The effective reproduction number (*R*_*t*_) fell below 1 when the average daily number of close contacts was reduced to ≤ 7 and quarantine/isolation was implemented on average at the same day of symptom onset in Guangzhou. Simulations showed that the outbreak could still be contained when quarantine is implemented on average 1 day after symptom onset while the average daily number of close contacts was reduced to ≤ 9 per person one week after the outbreak's beginning. Early quarantine and reduction of close contacts were found to be important for containment of the outbreaks. Early implementation of quarantine/isolation along with social distancing measures could effectively suppress spread of the Delta and more infectious variants.

## Introduction

As of September 30, 2021, severe acute respiratory syndrome coronavirus 2 (SARS-CoV-2), has resulted in more than 200 million coronavirus disease 2019 (COVID-19) cases and 4.8 million deaths worldwide. Since December 2020, several strains of SARS-CoV-2 were classified by the World Health Organization (WHO) as variants of concern (VOC). The Alpha (B.1.1.7) strain was first detected in the United Kingdom^1^, Beta (B.1.351) was first identified in South Africa^2^, and the Gamma (P.1) variant was initially documented in Brazil^3^. The Delta (B.1.617.2) strain was initially identified in India and is classified as a VOC^4^. Ongoing transmission of the Delta strain has been detected in 107 countries and regions worldwide until late 2021^5^. A study demonstrated that the B.1.617.2 variant has a higher rate of transmission than other variants, including the B.1.1.7 (Alpha) variant^6^. The Delta variant is characterized by spike protein mutations T19R, Δ157-158, L452R, T478K, D614G, P681R, and D950N^7^, which may affect immune responses directed toward key antigenic regions of the receptor binding protein (i.e., 452 and 478 sites) and contain deletions in part of the N-terminal domain^8^. Studies have reported that P681R is located at the S1/S2 cleavage site and suggest that these mutated strains may have increased replication, resulting in higher viral loads and increased transmission^9^. Meanwhile, mutations in the non-structural protein (NSP) of Delta variants may have an impact on viral transmission and pathogenicity. CoV-2 NSP6 inhibits autophagy, which is crucial for viral infections and induces cell death^10^. The mutation NSP6 T77A was commonly identified in Pakistani SARS-CoV-2 Delta variants during the fourth pandemic wave^11^. G671S in NSP12 identified in Delta has been reported to be emerging, increasing the protein's stability and maybe affecting its pathogenicity^11,12^. Furthermore, there was no significant difference in the clinical features between Delta and other strains^13^. The typical symptoms of a Delta infection were fever, dry cough, coughing with phlegm, weakness, shortness of breath, headache, and muscular aches. The majority of patients presented their initial symptoms of hypoesthesia or loss of smell and taste. Some patients with severe illness often have dyspnea or hypoxemia one week after disease onset, while others might rapidly develop acute respiratory distress syndrome (ARDS), septic shock, metabolic acidosis, coagulation dysfunction, and multiple organ failure. Extremely few cases show central nervous system involvement and acral ischemia necrosis.

**Figure 1:**
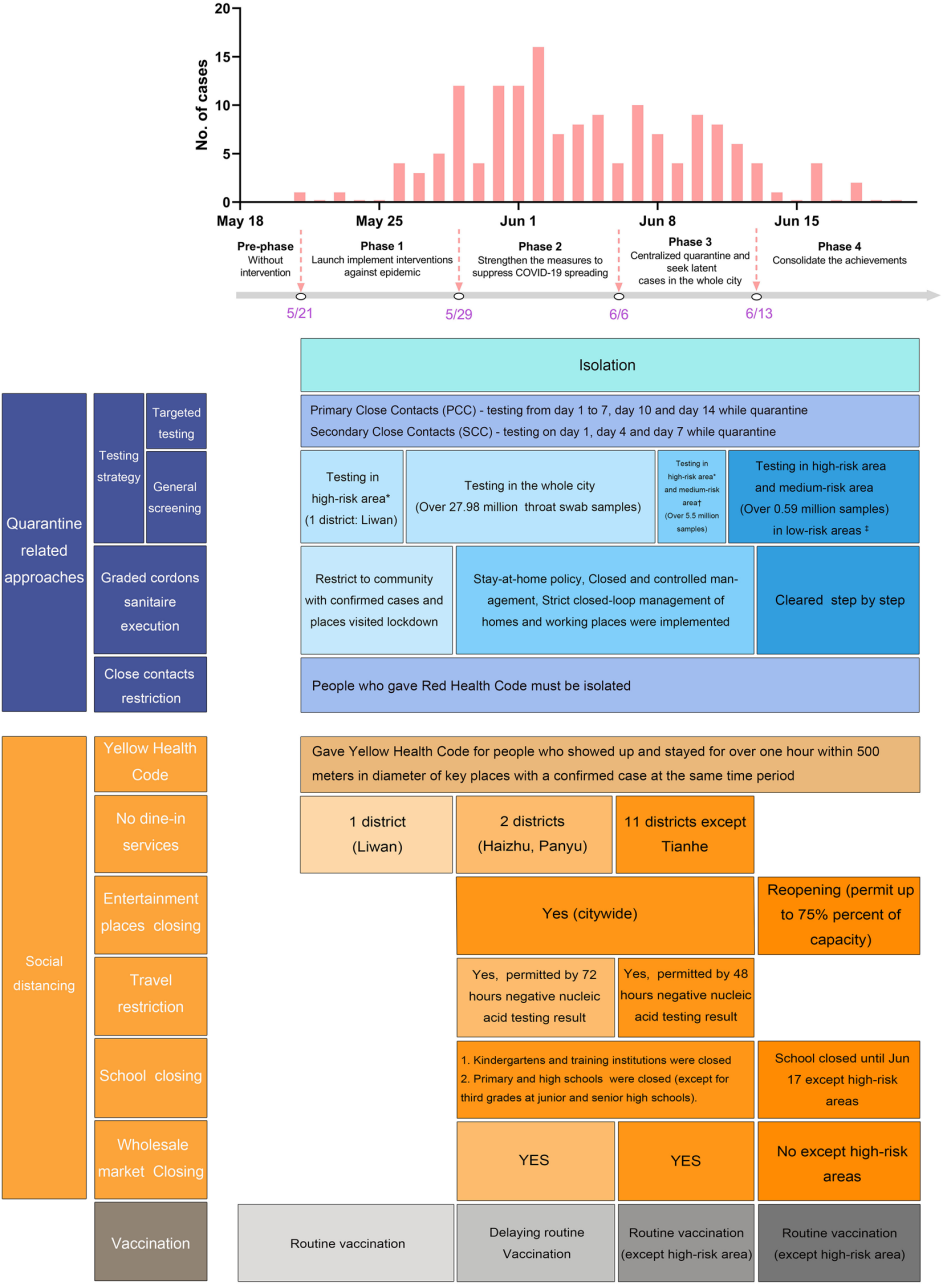
Trend of the epidemic curve and interventions across five periods during the Delta variant outbreak in Guangzhou.^*^The total number of infections with more than 50 cases, and a cluster of epidemics occurred within 14 days. ^†^The new infections within 14 days, and the total number of infections did not exceed 50; the total number of confirmed cases has exceeded 50, but no cluster of epidemics occurred within 14 days. ^‡^No confirmed cases were found for 14 consecutive days.

On May 21, 2021, the Delta variant was first isolated in Guangzhou, China during a local outbreak and confirmed using next generation sequencing (NGS). The outbreak was finally contained within a month, and the last COVID-19 case was reported on June 18, 2021; China adopted a containment strategy and implemented strict and timely non-pharmaceutical interventions (NPIs). Initially, citywide mass PCR testing was carried out to detect potential cases, with nearly 28 million throat swab samples collected from 13 million individuals by June 8. A red health code was generated for confirmed and suspected cases and their close contacts via smartphones for more precise and extensive contact tracing. Suspected COVID-19 cases and the close contacts of confirmed cases were all required to quarantine at home or in designated hotels to prevent virus transmission from symptomatic and asymptomatic cases. All confirmed cases were isolated immediately in designated hospitals. Additionally, other measures such as closure of schools and entertainment venues as well as travel restrictions were implemented on May 29, 8 days after the first case was detected (Fig. 1). Mask wearing was mandated. In mainland China, health code was used to apply mobility restrictions for individuals with possible exposure to SARS-CoV-2 infections. A yellow health code was generated for individuals who had remained within 500 m of a confirmed COVID-19 case for more than 1 h. A yellow health code will only change to a green health code after the person has taken a nucleic acid amplification test and obtained a negative result; until such time, they are required to maintain a safe physical distance from other people. No city-wide lockdown was implemented during the Guangzhou outbreak, but there were movement restrictions in some districts where cases were reported. Although these efforts and citywide precautionary measures were effective in this outbreak, the required level of control for the containment of the Delta variant has not been characterized. In addition, SARS-CoV-2 is an RNA virus, which is very susceptible to mutation and has the potential to develop a wide variety of subtypes due to its continual capacity for recombination and mutation^14^. For example, from late March to April 2021, a cluster of 37 COVID-19 patients in Japan, named "Cluster K," reported more severe illness^15^. The mortality rate has risen to 16.2%. Four of the mutations were detected on three nonstructural proteins (NSPs): one in nsp3 and nsp15, two in nsp6. A second one was found on the S protein. The model that we developed in this research may be used in the investigation of new SARS-CoV-2 subtypes in the future by altering parameters such as *R*_*0*_ and incubation period.

In this study, we aimed to understand the impact of various interventions on this outbreak by COVID-19 Delta strain as well as future outbreaks of more infectious strains, using a susceptible–exposed–infectious–recovered (SEIR) transmission model.

## Materials and Methods

### Construction of transmission dynamic model

**Figure 2:**
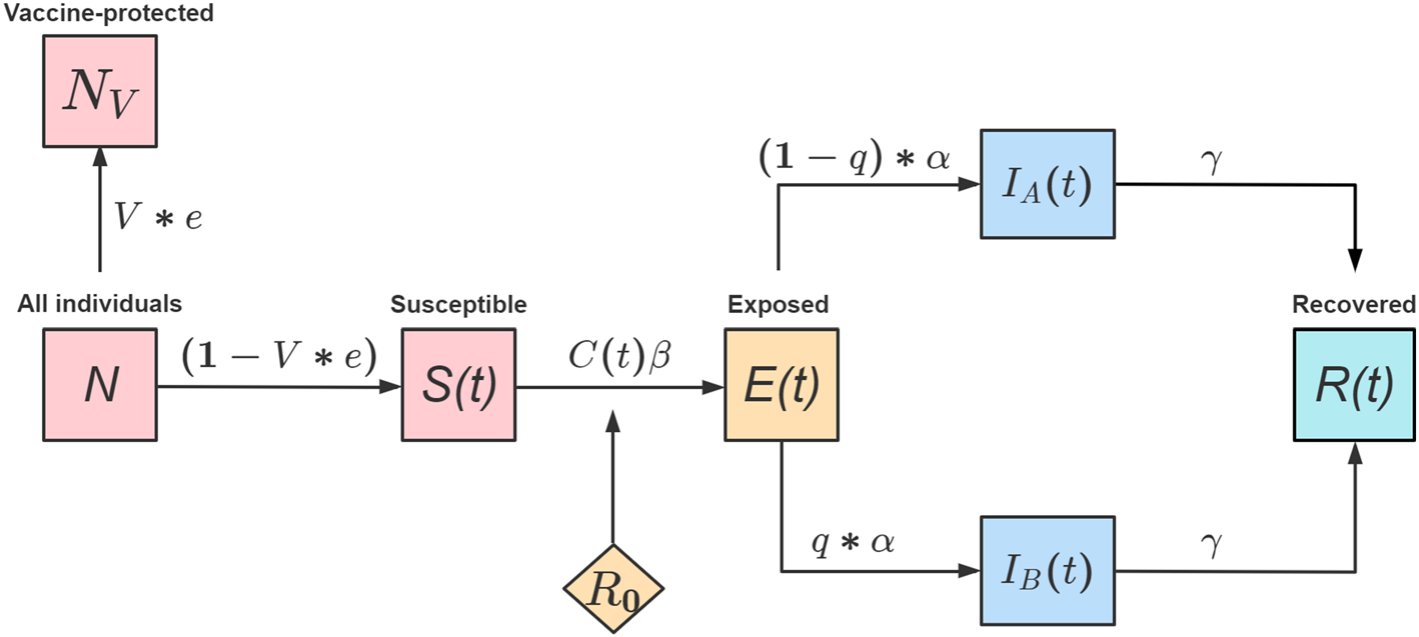
Schematic of the transmission model. *N*_*V*_: The number of unsusceptible people who got vaccinated and protected in Guangzhou; *V*: The number of people who were fully vaccinated (2 doses) before the outbreak in Guangzhou; *e*: Vaccine efficacy for preventing infection*; N*: The total population in Guangzhou*; S(t)*: The number of susceptible people in Guangzhou*; C(t)*: Average contact rate per active infectious person*; β*: The rate of transmission via contact with active infections; *E(t)*: The number of exposed people in Guangzhou*; q*: The quarantine/isolation effect (1/*t*_*c*_); *I*_*A*_*(t)*: The number of active infections (without quarantine/isolation); *I*_*B*_*(t)*: The number of quarantined/isolated infections; *R*_*0*_: The initial number of recovered people in Guangzhou; *R(t)*: The number of the recovery in Guangzhou; *α*: The rate of transmission for the exposed to be infected, which is equal to 1/L; *γ*: The rate of recovery, which is equal to 1/D; *I*_*A*_ can return to the *S* population and become infected.

We developed a modified SEIR model to model the spread of SARS-CoV-2 under different stages of control (Fig. 2). In this model, individuals were classified as vaccinated-protected (*N*_*V*_), susceptible (*S(t)*), close contact (*C(t)*), exposed (*E(t)*), infected without isolation (*I*_*A*_*(t)*), infected in quarantine/isolation (*I*_*B*_*(t)*), and recovered (*R(t)*). Specifically, susceptible individuals (*S(t)*) may become a close contact (*C(t)*) and shift to exposed individuals (*E(t)*) with an infection rate (*β*). Exposed individuals (*E(t)*) became infectious after the latent period. In this modeling study, we assumed that infectious individuals in quarantine/isolation (*I*_*B*_*(t)*) are completely isolated until recovery. We also assumed that recovered individuals (*R(t)*) will be immune and protected by neutralizing antibodies during the study period. To interrupt virus transmission, people with active infection should be isolated in a timely manner. We defined the time to control (*t*_*c*_) as the duration from exposure to quarantine/isolation. With a longer *t*_*c*_, people with active infection have more opportunities to spread the virus in the community. Due to the unobservable time of exposure, we used the symptom onset as the time of reference. If the date of quarantine was earlier than symptom onset, then *t*_*c*_ becomes negative. If quarantine/isolation can be implemented much earlier than symptom onset, it could cover most or all of the infectious period and hence more effective in preventing transmission. We use R statistical software (version.3.6.1) and Python 3.7 for all calculations and analyses.

Our modified model is given by:

1 $$\left\{ {\begin{array}{*{20}l} {\frac{{dS\left( t \right)}}{{dt}} = - \beta C(t)S(t)I_{A} (t)} \hfill \\ {\frac{{dE\left( t \right)}}{{dt}} = \beta C(t)S(t)I_{A} (t) - \alpha E(t)} \hfill \\ {\frac{{dI_{A} \left( t \right)}}{{dt}} = (1 - q)*\alpha E\left( t \right) - \gamma I_{A} \left( t \right)} \hfill \\ {\frac{{dI_{B} \left( t \right)}}{{dt}} = q*\alpha E(t) - \gamma I_{B} (t)} \hfill \\ {\frac{{dR\left( t \right)}}{{dt}} = \gamma I_{A} \left( t \right) + \gamma I_{B} (t)} \hfill \\ \end{array} } \right.$$

*S(t)*: The number of susceptible people in Guangzhou. *E(t)*: The number of exposed people in Guangzhou. *I*_*A*_*(t)*: The number of active infections (without quarantine/isolation). *I*_*B*_*(t)*: The number of quarantined/isolated infections. *R(t)*: The number of the recovery in Guangzhou. *N*: The total population in Guangzhou. *V*: The number of people who were fully vaccinated (2 doses) before the outbreak in Guangzhou. *e*: Vaccine efficacy for preventing infection. *L*: the mean latent period for the disease. *α*: The rate of transmission for the exposed to be infected, which is equal to 1/L. *β*: The rate of transmission via contact with active infections. *C(t)*: Average contact rate per active infectious person. *t*_*c*_: Mean time from onset to becoming infectious. *q*: The quarantine/isolation effect (1/ *t*_*c*_). *D*: Mean infectious period. *γ*: The rate of recovery, which is equal to 1/D. *S*_*0*_ : The initial number of susceptible people in Guangzhou is $N-{E}_{0}-{I}_{0}-{R}_{0}-V*e$. ${E}_{0}$: The initial number of exposed people in Guangzhou. ${I}_{0}$ : The initial number of infectious people in Guangzhou. ${R}_{0}$_:_ The initial number of recovered people in Guangzhou.

### Classification of five periods

To better present the dynamics of the Delta variant outbreak and corresponding interventions, the time without interventions from May 18 to 20 was considered as a pre-period and other four periods were defined on the basis of important interventions that could affect the spread of the strain in Guangzhou. The first period was from May 21 to 28, when routine control measures were taken. The local government first conducted surveillance through nucleic acid testing and restricted movement among confirmed cases and potential cases to the community in high-risk districts and confirmed cases were isolated. During the second stage (May 29 to June 5), the level of control measures was strengthened. The scope of nucleic acid testing was extended to the entire city and a quarantine strategy was formulated according to the different risk levels. Other social distancing interventions were also implemented, including in schools and places of entertainment, closing of wholesale markets, implementation of travel restrictions, and bans on dining services. A novel approach, the yellow health code, was generated on the mobile phone of any individual who had remained for at least 1 h within 500 m of a confirmed case. The third phase was between June 6 and 11 when several rounds of nucleic acid testing were conducted in all 11 districts of Guangzhou. Residents of the Baihedong subdistrict were transferred to a centralized quarantine hotel. After June 12 (considered the fourth phase), close contact tracing in high-risk areas and centralized quarantine were carried out by the government. More detail is provided in Fig. 1.

### Data source and model parameters

Cases of COVID-19, the number of close contacts and the time of quarantine/isolation were collected from the Center for Disease Control and Prevention (CDC) of Guangzhou. To improve interpretation of the SEIR model, we conducted a sensitivity analysis with respect to the different parameters. The model parameters primarily consisted of two categories. The first category comprised the characteristics of the Delta variant, with reference to Zhang, M. et al^16^, including the basic reproduction number (*R*_*0*_) and incubation period, among others. The second category of parameters were those associated with measures taken during the Guangzhou epidemic, such as the average close contact rate *C(t)* and effect of quarantine/isolation, among others. In subsequent sensitivity analysis, parameters such as the *R*_*0*_, incubation period, quarantine/isolation effect, and average contact rate were selected to evaluate the performance of different control strategies. The parameters in this model are given in Table 1.

**Table 1:** Epidemiological parameters.

Parameters		Values	Source	Sensitivity analysis
The proportion of people who got vaccinated 2 doses before epidemic in Guangzhou	$V$	13.64%	The People's Government of Guangzhou Municipality^17^	
The total population in Guangzhou	$N$	18.68 million	National Bureau of Statistics^18^	
Basic reproduction number	*R*_*0*_	3.2	Zhang, M. et al^16^, sensitivity analyses	3.2, 4.0, 5.0, 6.0 (Supplementary Figure S1)
Quarantine/isolation effect	$q$	0–100%	1/*t*_*c*_	
Vaccine efficacy for preventing infection by Delta variant	$e$	59%	Xiao-Ning Li. et al^19^	
Incubation period	$L$	4.4 days	Zhang, M. et al^16^. Duration from exposure to onset, sensitivity analyses	2, 3, 4.4, 5, 6 (Supplementary Figure S2)
The rate of transmission via contact with active Infections	$\beta$	0.0082	Total number of infections /total number of close contacts via infections	
Recovery period	*D*	14 days	Rees, E. M. et al^20^	10, 12, 14, 16, 18 days (Supplementary Figure S3)
Average contact rate	*C(t)*	Varied for each phase (0**–**31)		0-31 person × 10%, 20%, 30%, 40%, 50%, 60%, 70%, 80%, 90%, 100%, 110%, 120%, 130% (Supplementary Figure S4)
Time from onset of infectiousness to quarantine/isolation	*t*_*c*_	Quarantine: (-4**–**1 days) Isolation: (0–3 days)		− 3, − 2, − 1, 0, 1, 2, 3 days (Supplementary Figure S5)
Time of response to the whole control measure (phase I**–**IV) in Guangzhou	*T*	[3, 5, 7]		− 3, − 2, − 1, 0, 3, 5, 7 (Supplementary Figure S6)

With respect to the details of other parameters, the close contact rate, *C(t)*, is the average number of close contacts per person per day. According to the CDC, the average *C(t)* in the five phases (Pre, I, II, III, IV) was 31, 29, 7, 7, and 0, respectively (Supplementary Table S1). Additionally, we used the parameter *t*_*c*_ in the evaluation of quarantine/isolation measures. The average period from quarantine/isolation to onset during the I–IV consecutive phases of the Guangzhou outbreak was 0.88 day, − 0.26 day, − 1.8 days and − 4.75 days (Supplementary Table S2), respectively. As mentioned above, the quarantine is significantly effective when initiated earlier during the incubation period, before symptom onset. Therefore, the parameter *t*_*c*_ is the sum of the incubation period and the difference between the start time of the quarantine/isolation measure and time of symptom onset. Moreover, the parameter *t*_*c*_ has an inverse relationship with the effect of quarantine/isolation. With a shorter *t*_*c*_, the quarantine/isolation measure is implemented swiftly. This means that the number of active infections is likely to be sufficiently reduced as to interrupt virus transmission. To evaluate outcomes with a delay in implementation of the whole control measure (phase I–IV) in Guangzhou, the parameter *T* was used in this model. The parameter *T* is assumed to be 3, 5, 7 days if the delayed measures for the specific period have a significant impact on the total infections or not.

### Sensitivity analyses

To explore the influence of different parameters in the prediction model, we analyzed the sensitivity of the initial transmission rate (*R*_*0*_), incubation period (*L*), recovery period (*D*), the time of response to control measures (phase I–IV) in Guangzhou (*T*), the time from onset to quarantine/isolation (*t*_*c*_) and close contact rate *C(t)* in the model. The ranges of these parameters used for sensitivity analysis are shown in Table 1. The features of the Guangzhou pandemic are used in the study to determine the baseline scenario, which is shown in Table 1. Considering that variation in transmissibility, we changed *R*_*0*_ (4.0, 5.0, 6.0) and incubation period (*L*) (2, 3, 5, 6 days) to evaluate the outbreak scale of the mutant virus under the different control conditions of the Delta mutant strain. Moreover, since the strength of NPIs can have a variable effect on the outbreak size of Delta variants, we altered the time of response to control measures (phase I–IV) in Guangzhou (*T*), time from onset to quarantine/isolation (*t*_*c*_) and close contact rate *C(t)* to assess the effectiveness of NPIs for Delta variant. It is speculated that shorter *T*, *t*_*c*_ and fewer *C(t)* would lead to lower number of cases. We conducted sensitivity analysis by changing the value of one or more parameters.

### Simulation scenarios

Considering how implementation of control measures affected the SARS-CoV-2 outbreak, we assessed the total infections, new infections, and effective reproductive number (*R*_*t*_) under three simulation scenarios. In the first scenario, considering the impact of timely implementation of the whole control measures in Guangzhou, the time to implement the interventions was postponed by 3 days and 5 days, respectively. In the second scenario, considering the impact of timeliness of quarantine/isolation implementation on the epidemic, the time to quarantine/isolation was advanced by 2 days and delayed by 2 days and by 3 days. In the third scenario, we considered that the effectiveness of social distancing measures is reflected by the average number of close contacts per person per day. We altered the close contact rate *C(t)* by setting it to increase by 10% and 20% and to decrease by 10% and 20%. All results were compared with and without the interventions implemented in Guangzhou during the SARS-CoV-2 Delta variant outbreak. The model with Guangzhou interventions was set as a base scenario. When the threshold of new cases is fewer than one patient per day within 40 days, the epidemic is considered to be contained before 26th June;

Owing to the ongoing evolution of SARS-CoV-2, additional infectious variants could emerge in the future. In a fourth simulation scenario, we changed the initial transmission rate *R*_*0*_ (4, 5, and 6) and incubation period (2, 4, and 6 days) of the virus with implementation of the measures taken in Guangzhou. We then evaluated the total number of confirmed cases under different implementation times during the whole period II–IV and various quarantine/isolation times to identify a suitable combination of control measures to effectively slow an outbreak caused by a SARS-CoV-2 variant with an *R*_*0*_ = 6 and an incubation period of 2 days.

**Figure 3:**
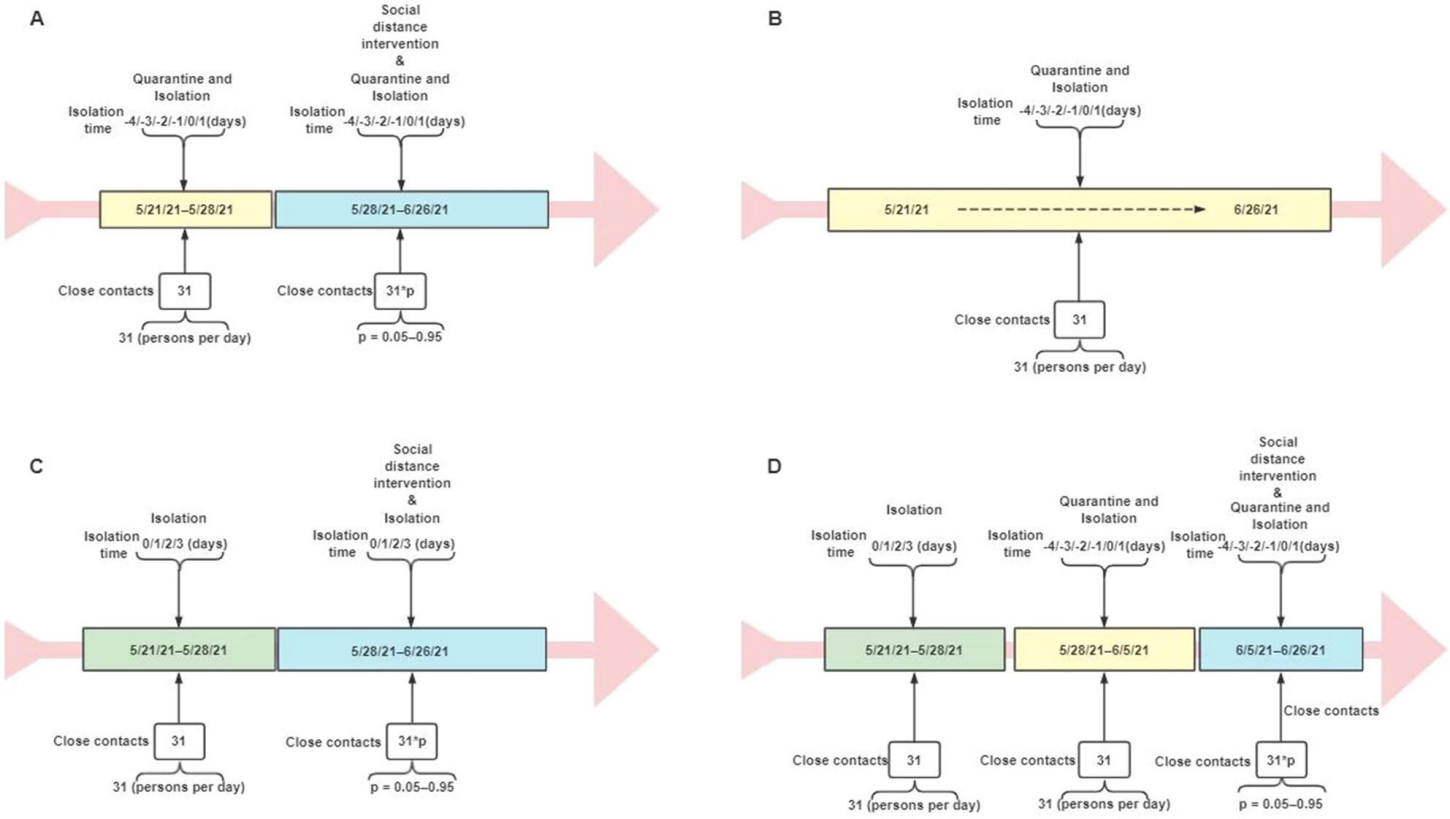
Parameters of the four strategies. (**A**) Strategy 1. Initial quarantine/isolation followed by social distancing. We assumed the time to quarantine/isolation was − 4 to 1 days from May 21 to June 26, owing to mass testing and contact tracing. Considering that the first case was found and the implementations were imposed by government in Guangzhou from May 21, we calculated 31 daily contacts per person in Guangzhou prior to that date as the routine setting. When social distancing was imposed from May 28 to June 26, 2021, we used 31 multiplied by various percentages (5–95%) to indicate the effect of the intervention. (**B**) Strategy 2. Quarantine/isolation only. We assumed the time to quarantine/isolation was − 4 to 1 days and the number of daily contacts per person was 31 from May 21 to June 26. (**C**) Strategy 3. Initial isolation followed by social distancing. Considering that positive cases could not be identified in a timely manner at symptom onset, we assumed that the time to isolation was 0–3 days from May 21 to June 26. When social distancing was adopted from May 28 to June 26, we used 31 multiplied by various percentages (5–95%) as the effect of the intervention, according to stringency. (**D**) Strategy 4. Initial isolation followed by quarantine and social distancing. This strategy was divided into three phases. Isolation was implemented and set to 0–3 days in phase I (May 21–28). Quarantine was imposed in phase II (May 28–June 26) and changed to − 4 to 1 days. Social distancing was implemented in phase III (June 5–June 26); we used 31 daily contacts per person multiplied by different percentages (5–95%) to simulate the effect of these control measures.

Additionally, four strategy scenarios were proposed for implementation. The first strategy involved quarantine, isolation, mass testing, contact tracing on the day that the first case was identified, then implementation of a series of social distancing interventions 8 days after the first measure taken, as in the pattern of intervention followed in Guangzhou. The second strategy was only quarantine, isolation, mass testing, and contact tracing on the day the first case was detected. The third strategy was isolation during the day the first case was found, and social distancing implemented 8 days after the isolation imposed. The fourth strategy was isolation during the day the first case was detected, quarantine, mass testing, contact tracing 8 days after the first measure implemented and social distancing intervention implemented 7 days after the second measure taken. The parameters of the four strategy scenarios are shown in Fig. 3. The effects of mass testing and contact tracing were implicitly modeled by the reduction in days to be isolated. To differentiate between Isolation and Quarantine, the duration of Isolation was set to 0/1/2/3 day, while the duration of Quarantine was set to − 4/− 3/− 2/− 1/0/1 day.

When analyzing the differences across four intervention scenarios ((i). impact of timely implementation of the control measures in Guangzhou; (ii). the impact of timeliness of quarantine/isolation implementation; (iii). the effectiveness of social distancing measures; (iv). ongoing evolution of SARS-CoV-2), we compared the cumulative and new infections, *R*_*t*_ of each scenario and 95% confidence interval (CI). When exploring differences among four strategy scenarios (Fig. 3), we compared the median cumulative number of cases in each simulation. We calculated the interquartile range (IQR) as the 25th and 75th simulation of the cumulative cases at 40 days. Moreover, when estimating the effectiveness of interventions across infectivity scenarios, we estimated the cumulative infections and 95% confidence interval (CI) in each simulation. The CIs was calculated by mean ± 1.96*standard deviation.

## Results

**Figure 4:**
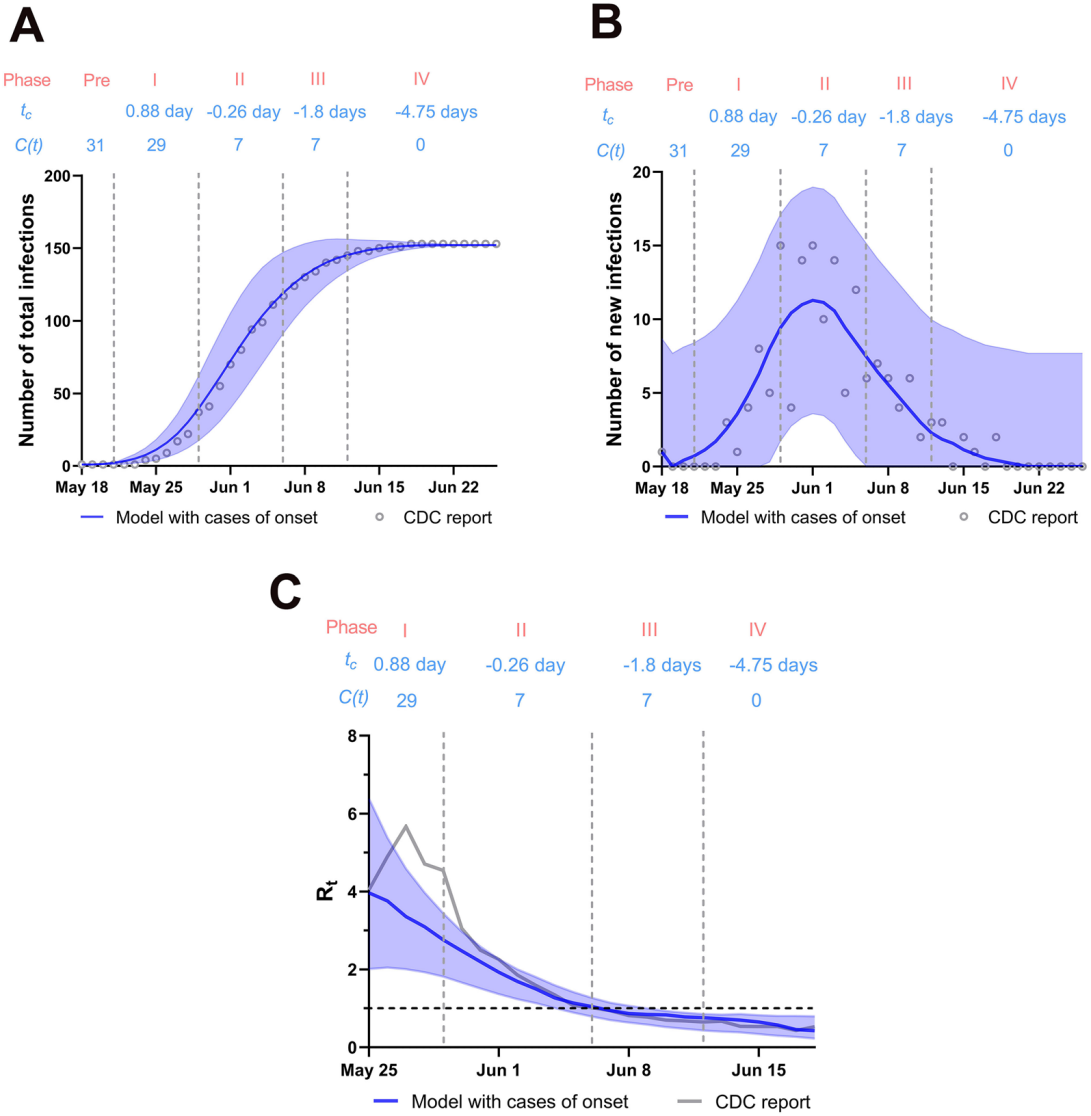
Cumulative infections, new confirmed cases, and *R*_*t*_ according to the model and CDC reported data. (**A**) Cumulative infections; (**B**) new confirmed cases; (**C**) *R*_*t*_*;* Blue shaded areas show the 95% CI for simulation with case of onset. *t*_*c*_: time from onset of infectiousness to quarantine/isolate; *C(t)*: Average contact rate.

Our model simulated the trend of the SARS-CoV-2 Delta variant outbreak in Guangzhou, China with strict interventions from May 18, 2021 to June 26, 2021 (40 days). The outbreak size of the model was 152 infections (95% CI: 152**–**153), and the timing of the peak was June 1, corresponding to 153 confirmed cases overall on June 25, as reported by the CDC. Our model showed that the *R*_*t*_ dropped below 1.0 on June 6, 2021 in agreement with June 6, 2021, as reported by the CDC (Fig. 4). The average times between symptoms onset and quarantine in different phases were calculated, 0.88 day in phase I, − 0.26 day in phase II, − 1.8 days in phase III, − 4.75 days in phase IV. The average number of close contacts per confirmed case daily was estimated, 31 in pre-phase, 29 in phase I, 7 in phase II, 7 in phase III, 0 in phase IV.

**Figure 5:**
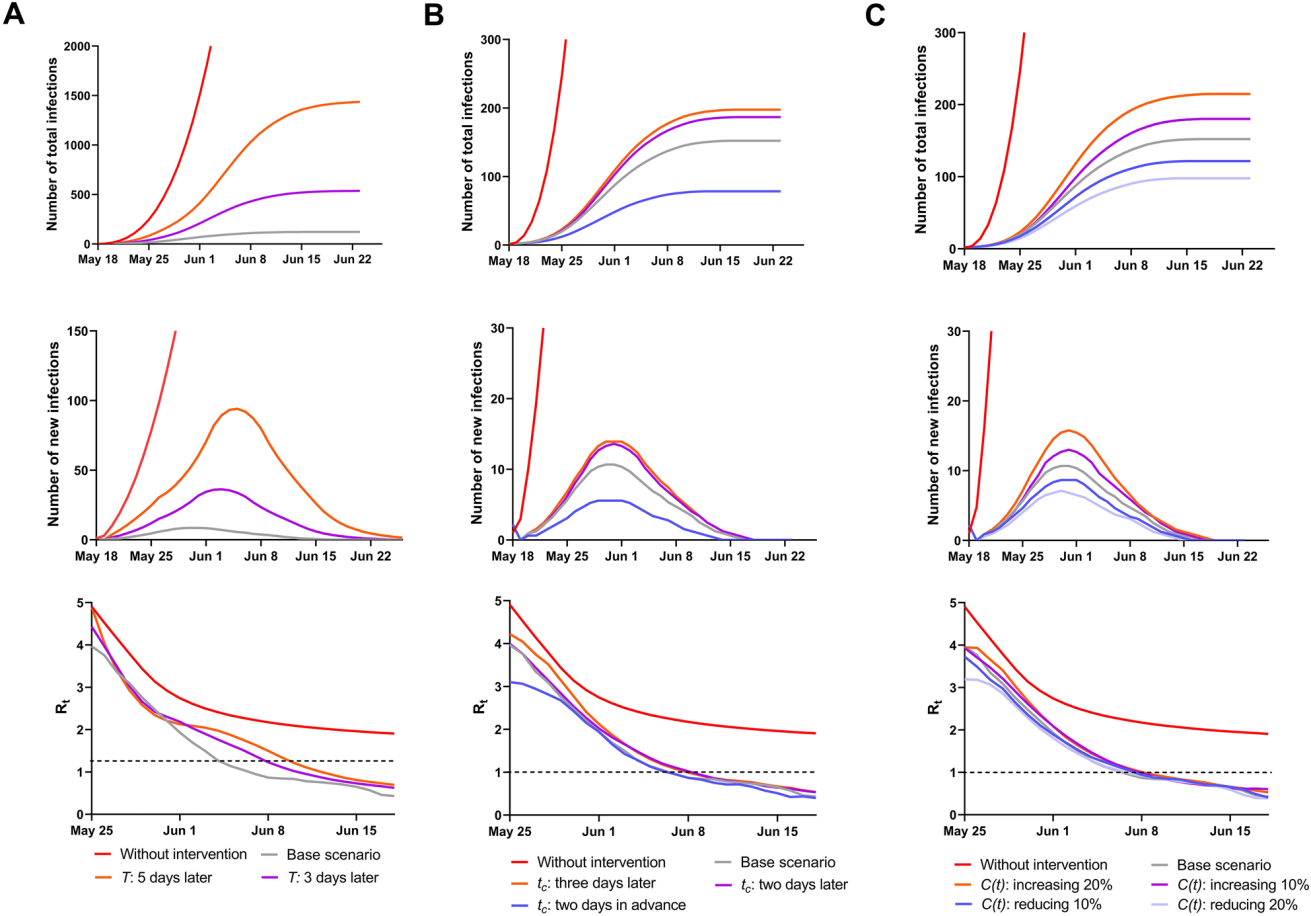
Estimation of effectiveness of Guangzhou interventions under three scenarios. (**A**) timely implementation of control measures (**B**) timeliness of quarantine (**C**) percent change in close contacts owing to social distancing. Base scenario: model with Guangzhou interventions; *T*: Time of response to t whole control measure (phase I**–**IV) in Guangzhou; *t*_*c*_: time from onset of infectiousness to quarantine/isolation; *C(t)*: Average contact rate.

Initially, our model predicted the total number of confirmed cases without any interventions to be 29,853 cases (95% CI: 21,088–38,618) in 40 days, shown in Fig. 5. However, when the first case was identified on May 21, the Guangzhou government rapidly launched emergency measures in response. Quarantine-related approaches such as mass nucleic acid testing to search for additional cases and lockdown in high-risk areas were adopted. After 8 days, the elapsed time between symptom onset and quarantine, isolation was maintained at 0.88 day, on average; thus, the trend of the epidemic was in decline. However, a peak did not occur. As the range of the outbreak extended, a quarantine strategy was established in various stages and social distancing interventions (i.e., in schools and places of entertainment, closing of wholesale markets) were implemented. According to our model, if measures were implemented 3 days later, the cumulative number of cases would be 537 (95% CI: 532–542), which is 4.4 times higher than the number with the Guangzhou intervention; the timing of the peak would be delayed by 4 days. If the control interventions were taken 5 days later, the total number of infections would be 1446 (95% CI: 1428–1465), which was 11.9 times greater than with the Guangzhou interventions; the timing of the peak would be delayed 6 days. Consequently, we found that the timing of implementing public health measures played a vital role in ending the outbreak of the SARS-CoV-2 Delta strain in Guangzhou, which could influence the ending timepoint of the epidemic.

Because the quarantine strategy was a primary measure of control in this epidemic, we estimated whether the outbreak size could be changed using contact tracing if some level of elapsed time between symptom onset and quarantine were maintained. Owing to the mean incubation time of the virus 4.4 days, it is necessary to quarantine potential confirmed cases early to prevent household and community transmission (Fig. 5B). If an infected patient were quarantined 2 days earlier, according to Guangzhou interventions, the rate of confirmed cases could be reduced to 48.7% and the peak occurring 1 day before. On the contrary, if infected individuals were quarantined 2 or 3 days later, the infection rate would increase to 23.0% and 30.3% and the peak occur nearly base scenario, respectively. The aim of quarantine is to identify additional confirmed and suspected cases as far as possible. Initially, mass testing was implemented in high-risk areas of Guangzhou and even in the entire city during phases I and II, with over 27.98 million throat swabs collected. According to the distribution of infections, the different risk levels were assessed by public health professionals and quarantine measures were divided into a stay-at-home policy, closed and controlled management, and closed-loop management in homes and workplaces, so as to maintain as much functioning of society as possible. When primary and even secondary close contacts were quarantined, they were required to be tested every few days. Additionally, a red health code, which a indicates confirmed case or one with a high level of suspicion, was generated on the mobile phone of individuals and their close contacts to inform them that they must isolate. According to the timeliness of quarantine, confirmed COVID-19 cases were quarantined before symptom onset in the early stages of the outbreak.

We also considered whether social distancing interventions were effective in containing the spread of the virus. We used the close contact rate to indicate the effectiveness of social distance interventions. With stricter and more widespread implementation of social distancing, the rate of close contact was further reduced. Our results showed that the social distancing interventions in phase II reduced the close contact rate by 77.4%. Thus, we estimated that if some levels of close contact were maintained, the outbreak size could change (Fig. 5C). For example, if daily contact in other settings (i.e., outside the home, at work, and in school) decreased to 20%, the cumulative number of infections would be reduced by 35.5%. We then estimated that daily contacts would rise to 20% if the measures were relaxed, with the total number of cases reaching 41.5%. The peak of new cases of both levels of daily contacts occurred close to the base scenario Therefore, the potential impact of social distancing interventions is on the outbreak size and should not be ignored.

**Figure 6:**
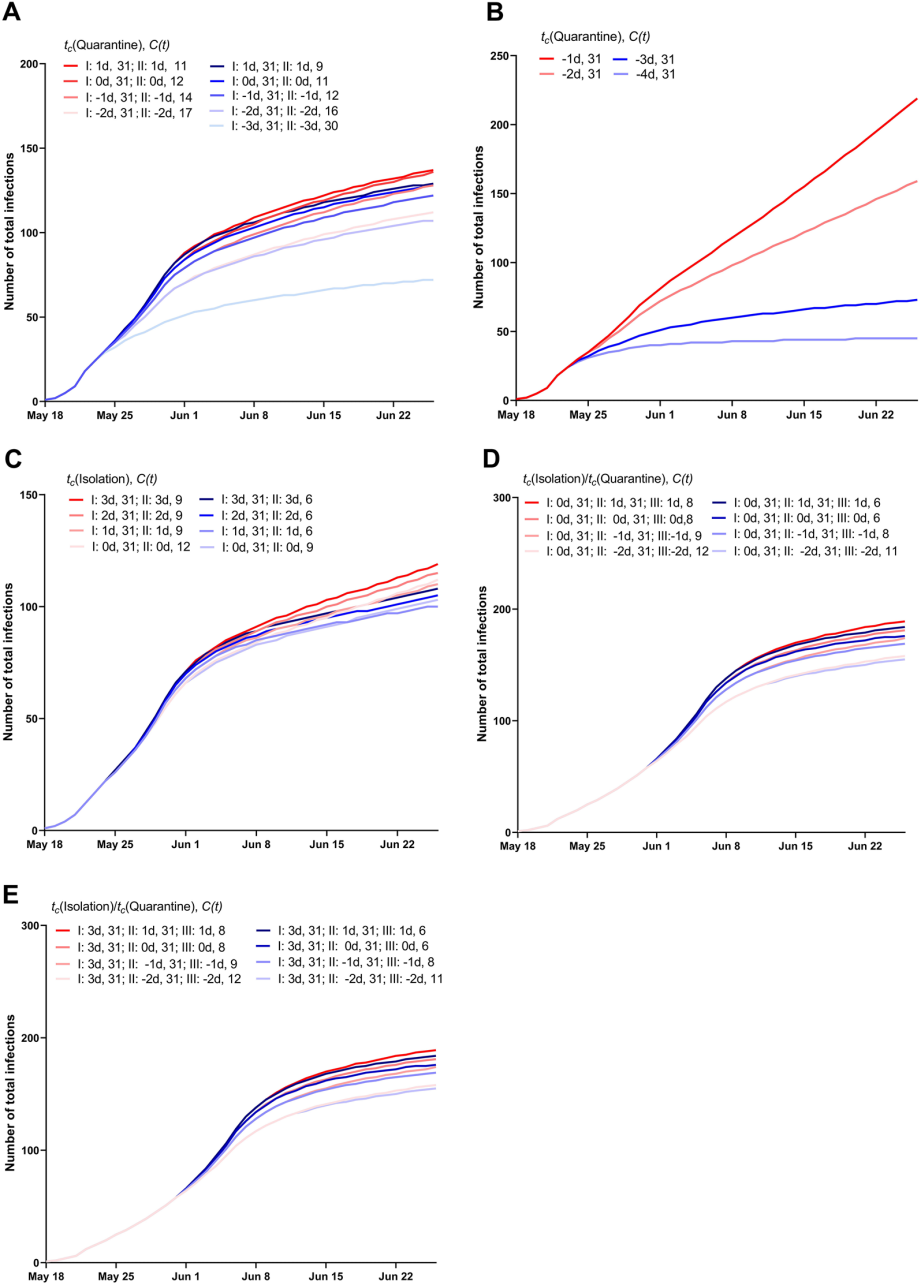
Estimation of effectiveness of the four strategies. (**A**) Strategy 1: initial quarantine/isolation followed by social distancing, including two phases. The first phase is from May 21 to May 28: *t*_*c*_(Quarantine) = − 4d, − 3d, − 2d, − 1d, 0d, 1d, *C(T)* = 31; The second phase is from May 28 to June 26: *t*_*c*_(Quarantine) = − 4d, − 3d, − 2d, − 1d, 0d, 1d, *C(T)* = 31*(5–95%); (**B**) Strategy 2: quarantine/isolation only from May 21 to June 26: *t*_*c*_(Quarantine) = − 4d, − 3d, − 2d, − 1d, 0d, 1d, *C(T)* = 31; (**C**) Strategy 3: initial isolation followed by social distancing, including two phases. The first phase is from May 21 to May 28: *t*_*c*_(Isolation) = 0d, 1d, 2d, 3d, *C(T)* = 31; The second phase is from May 28 to June 26: *t*_*c*_(Isolation) = 0d, 1d, 2d, 3d, *C(T)* = 31*(5–95%); (**D**) Strategy 4: initial isolation followed by quarantine and social distancing. The isolation was initially implemented the same day of symptom onset, including three phases. The first phase is from May 21 to May 28: *t*_*c*_(Isolation) = 0d, *C(T)* = 31; The second phase is from May 28 to June 5: *t*_*c*_(Quarantine) = − 4d, − 3d, − 2d, − 1d, 0d, 1d, *C(T)* = 31; The third phase is from June 5 to June 26: *t*_*c*_(Quarantine) = − 4d, − 3d, − 2d, − 1d, 0d, 1d, *C(T)* = 31*(5–95%); (**E**) Strategy 4: initial isolation followed by quarantine and social distancing. The isolation was initially imposed on average 3 days after symptom onset, including three phases. The first phase is from May 21 to May 28: *t*_*c*_(Isolation) = 3d, *C(T)* = 31; The second phase is from May 28 to June 5: *t*_*c*_(Quarantine) = − 4d, − 3d, − 2d, − 1d, 0d, 1d, *C(T)* = 31; The third phase is from June 5 to June 26: *t*_*c*_(Quarantine) = − 4d, − 3d, − 2d, − 1d, 0d, 1d, *C(T)* = 31*(5–95%); Red line indicates that the threshold of new cases is more than one per day within 40 days, suggesting that the epidemic cannot be controlled before 26th June; Blue line means that the threshold of new cases is fewer than one patient per day within 40 days, showing that the epidemic can be controlled before 26th June; “I”: the first phase; “II”: the second phase; “III”: the third phase.

We considered a total of four different strategies. In the first strategy (Fig. 6A), quarantine, isolation, mass testing, and contact tracing were implemented in the early stage of the outbreak; social distancing was then implemented 8 days after the first COVID-19 case was detected. We found that the outbreak would end as long as cases were quarantined on average within 3 days before symptom onset despite maintaining 30 close contacts per day. Moreover, the epidemic would also be contained with time from symptom onset to quarantine − 2 days and maintaining 16 close contacts per person per day or time from symptom onset to quarantine − 1 day and 12 daily close contacts or quarantined on average at the same day of symptom onset with an average of 11 close contacts per day. The median cumulative number of infections on day 40 was only 79 cases (IQR: 45–163). In Guangzhou, we found that the effective reproduction number (*R*_*t*_) fell below 1 when the average daily number of close contacts was reduced to ≤ 7 cases (Fig. 4C) and quarantine/isolation was implemented on the same day of symptom onset, consistent with strategy 1. The second strategy was quarantine, isolation, mass testing, and contact tracing on the day the first case was detected, and the median cumulative number of infections on day 40 was 152 cases (IQR: 42–291). The outbreak would end if positive cases were quarantined 4 and 3 days before the onset of symptoms (Fig. 6B). Compared with strategy 1, strategy 2 lacked the impact of social distance interventions, suggesting that quarantine alone is insufficient when positive cases were quarantined less than 2 days before the onset of illness. The third strategy was isolation implemented in the early stage of the outbreak and then social distancing implemented 8 days after isolation, with an estimated median cumulative number of cases on day 40 of 253 (IQR: 87–637). Reducing the average number of close contacts to 6 people per day led to the outbreak ending despite positive cases were isolated 3 days after the onset of symptoms (Fig. 6C). Finally, the fourth strategy consisted of three phases: isolation first implemented on May 21 (phase I) and then quarantine 8 days later (phase II), followed by social distancing (phase III). The outbreak would be contained, as long as confirmed cases were initially isolated on average within 3 days after symptom onset in phase I, were isolated on average within 1 days after symptom onset in phase II and the average number of close contacts were less than 6 people per day in phase III (Fig. 6D,E). However, the ending time point would be delayed. The median cumulative number of infections was 329 cases within 40 days (IQR: 63–1411). Overall, based on the lowest median infection rate, the first strategy could potentially be effective in substantially reducing the outbreak size to combat outbreaks of the SARS-CoV-2 Delta variant.

Owing to the ongoing evolution of SARS-CoV-2, it is likely that novel strains with greater transmissibility and stronger binding affinity and possibly decreased antibody neutralization will emerge in the future. Thus, we modeled the Guangzhou intervention to test various levels of *R*_*0*_ and incubation periods of SARS-CoV-2. We found that the outbreak size of a variant with *R*_*0*_ = 6 and an incubation period of 2 days was as high as 721 (95% CI: 743–779) cases if following the Guangzhou strategy (Table 2). Compared with an assumption of no interventions for Delta (breakout size: 29,853 cases (95% CI: 21,088–38,618), strategy 1 implemented in Guangzhou still had a significant impact on reducing the total number of infections. We also adjusted the timing of implementing social distancing measures and the timeliness of quarantine to establish a better strategy. The results are shown in Table 3. Our model showed that based on parameters of the Guangzhou intervention and a more infectious variant with *R*_*0*_ = 6 and a 2-day incubation period, an outbreak would be well controlled by adjusting the timing of social distancing to begin 2 or 3 days earlier.

**Table 2:** Outbreak size using Guangzhou interventions for more threatening SARS-CoV-2 variants.

Parameter	Outbreak size
*R*_*0*_ = 6, incubation = 2	721 (95% CI: 703–739)
*R*_*0*_ = 6, incubation = 4	333 (95% CI: 325–341)
*R*_*0*_ = 6, incubation = 6	218 (95% CI: 213–223)
*R*_*0*_ = 5, incubation = 2	564 (95% CI: 549–579)
*R*_*0*_ = 5, incubation = 4	271 (95% CI: 265–277)
*R*_*0*_ = 5, incubation = 6	176 (95% CI: 172–178)
*R*_*0*_ = 4, incubation = 2	423 (95% CI: 411–435)
*R*_*0*_ = 4, incubation = 4	212 (95% CI: 207–215)
*R*_*0*_ = 4, incubation = 6	139 (95% CI: 135–142)
*R*_*0*_ = 3.2, incubation = 4.4 (Delta variant)	152 (95% CI: 149–155)

**Table 3:** Top ten cumulative number of interventions for a variant with *R*_*0*_ = 6 and incubation period of 2 days, altering the timing of social distancing (phases II, III, IV) and timeliness of quarantine based on the Guangzhou parameters.

Top 10	Timing (*T*) of social distancing (days after symptom onset of the first case)	Quarantine (*t*_*c*_): timeliness of quarantine (day)	(*T*, *t*_*c*_)	Outbreak size	Ending time point
Baseline	11	I: 0.88; II: − 0.26; III: − 1.8; IV: − 4.75	(0, 0)	708 (95%CI: 704–712)	26 days
1	8	I: − 2.12; II: − 3.26; III: − 4.8; IV: − 7.75	(− 3, − 3)	5 (95%CI: 3–7)	0 days
2	8	I: − 1.12; II: − 2.26; III: − 3.8; IV: − 6.75	(− 3, − 2)	14 (95%CI:12–16)	12 days
3	9	I: − 2.12; II: − 3.26; III: − 4.8; IV: − 7.75	(− 2, − 3)	25 (95%CI:23–27)	15 days
4	8	I: − 0.12; II: − 1.26; III: − 2.8; IV: − 5.75	(− 3, − 1)	25 (95%CI:22–28)	15 days
5	8	I: 0.88; II: − 0.26; III: − 1.8; IV: − 4.75	(− 3, 0)	32 (95CI:29–35)	16 days
6	8	I: 1.88; II: 0.74; III: − 0.8; IV: − 3.75	(− 3, 1)	38 (95CI:34–42)	17 days
7	8	I:2.88; II: 1.74; III: 0.2; IV: − 2.75	(− 3, 2)	43 (95%CI:40–48)	17 days
8	10	I: − 2.12; II: − 3.26; III: − 4.8; IV: − 7.75	(− 1, − 3)	43 (95%CI:37– 46)	20 days
9	8	I: 3.88; II: 2.74; III: 1.2; IV: − 1.75	(− 3, 3)	47 (95%CI:46–48)	18 days
10	9	I: − 1.12; II: − 2.26; III: − 3.8; IV: − 6.75	(− 2, − 2)	63 (95%CI:57–68)	20 days

## Discussion

The COVID-19 pandemic has led to huge global social and economic loss and the rollout of vaccination has helped to reduce its impact. However, there was a large variation in vaccination coverage across regions and breakthrough infections were also reported for the emerging SARS-CoV-2 variants. Therefore, it is still necessary to maintain at least some of the NPIs to control SARS-CoV-2 transmission.

Our analysis suggests that NPIs such as those implemented in Guangzhou during a Delta variant outbreak in May 2021, which included early quarantine/isolation followed by other social distancing measures (strategy 1), were able to contain the spread of Delta and likely other emerging variants with higher transmissibility. As simulated by our model, the average daily number of close contacts was reduced to ≤ 7 cases in phase II was critical for ending the outbreak. Stringent social distancing can reduce the number of close contacts. In Guangzhou, the average number of close contacts was reduced from over 31 to 7 by various social distancing measures which have a strong impact on containing the epidemic.

In our study, we showed that timeliness of quarantine has helped to slow down the epidemic. Early quarantine of close contacts was a possible with ability of performing mass scale of testing. At the beginning of the outbreak in Guangzhou, mass testing was performed only in high-risk areas. When a few positive cases began to be reported in other non-high risk areas, mass testing was expanded to the entire city. If mass testing in the whole city was implemented earlier, the outbreak size would have been smaller. Not only in China, mass testing was also used in other counties, such as United Arab Emirates, Slovakia^21,22^. While pre-symptomatic transmission plays an important role in the transmission of COVID-19^23^, quarantine and isolation measures were supported by mass and frequent PCR testing, and stringent contact tracing in Guangzhou. Under such conditions, symptom presentation may have a limited impact on quarantine or isolation. Overall, our simulation suggested that similar strategies are likely effective for potential variants with higher and faster transmissibility.

However, considering the potential costs of the control strategies, the choice of them would be important. The findings indicate that strategy 1 (quarantine with social isolation) is the most effective. The major difference between the four strategies is whether quarantine measures and social distancing measures are used and when they are implemented. Comparing strategy 1 to strategy 4, the sooner quarantine measures and social distancing measures are implemented, the sooner the epidemic will be contained and the lower the median cumulative number of infected people will be affected. If only a single quarantine or social distancing (as seen in Fig. 6B,C) is used, the parameters for the isolation days and the average number of close contacts will be stricter, resulting in more restrictive controls. However, it is a practical concern if the strict NPIs would have a significant negative impact on the society and economy. First, the sooner effective viral control measures are implemented, the fewer patients will be infected and died, and there is a considerable association between this phenomenon and the rise in GDP growth rate. This idea is consistent with other studies^24,25^. In addition, research has proven that stringent social distance measures are a required and effective NPI. Chen et al.^26^ developed a model and estimated that the medical expenses paid by the United States during the first wave of the pandemic were one trillion dollars, but could be drastically reduced to thirty-five billion dollars if strong social distancing measures were implemented. Similarly, Brzezinski et al.^27^ examined the medical and economic expenses associated with COVID-19 and determined that in a non-lockdown situation, the cost would be 16.1% of annual GDP per capita, but in a lockdown scenario, it will cost 15.2% of the per capita yearly GDP. The results demonstrate that tight rules do not place a significant strain on the economy, but rather cut medical expenses. Australia and Indonesia come to similar conclusions based on their research^28,29^. Moreover, human life has great worth. If the outbreak causes a substantial number of fatalities, it will be another severe blow to the social economy. Therefore, managing the epidemic as quickly as possible and avoiding human infection and mortality are crucial for averting economic decline. Based on our findings, the most effective strategies are quarantine and social distancing. If they are applied as quickly as possible at the beginning of an outbreak, the measures may be relaxed earlier, the time required to control the epidemic can be shortened, and the number of fatalities can be reduced. In conclusion, it would not significantly harm the economy.

In light of the high transmissibility and harmful of the Delta variant, a number of studies have investigated the control measures for the Delta variant, with the goal of providing a reference for governments throughout the world in their efforts to manage the pandemic. Nguyen et al. analyzed the efficiency of control measures using *Rt* values and the forecasted scale of the epidemic, providing support for Vietnam's "Zero COVID" policy and confirming that the Vietnamese government's approach was successful^30^. Likewise, the Chinese government follows the zero COVID-19 policy. The study also used *Rt* values and epidemic simulation to assess the effectiveness of the control measures implemented during the pandemic in Guangzhou and is devoted to assisting China with its epidemic control. In addition, there are articles that simulate scenarios and provide guidance on the containment of the Delta variant. With social distance parameters, Chang et al. simulated the scenarios of centralized isolation, home isolation, and school closures in Australia using a re-calibrated agent-based model. As long as ≥ 70% of the population was isolated at home, the epidemic could be successfully managed^31^. Also in Australia, Lasser et al. used an agent-based epidemiological model in conjunction with local vaccination to quantify the effect of NPIs (such as reducing the number of students in the classroom, wearing masks, and isolating at home) and to suggest reasonable control measures for school attendance. In addition to immunization, they discovered that schools need more than two NPIs to properly limit the epidemic^32^. Moreover, Layton et al. investigated the impact of NPIs on the Ontario, Canada epidemic using the optimized Susceptible Infection Recovered type model and discovered that the third vaccination and stringent NPIs may prevent the spread of VOC^33^. Compared with them, our study aimed to evaluate the effectiveness of measures against the epidemic in Guangzhou by calculating the Rt value and using the optimized transmission dynamics model. The simulation of different policy tightening scenarios is also conducted using the average number of close contacts and the isolation days. Even in the face of an unknown subtype of the SARS-CoV-2, it is possible to simulate the effectiveness of these measures by our model.

At present, the Omicron variant has spread to more than 100 nations and regions throughout the globe owing to its high transmissibility and immune escape, becoming the main variant^34^. Brian J. Willett et al. discovered significant neutralization resistance by Omicron BA.1 and BA.2 variants in vitro using sera from individuals immunized with ChAdOx1, BNT162b2, and mRNA-1273. Meanwhile, the Omicron variants BA.1 and BA.2 did not produce cell syncytia in vitro and favored an endosomal entry mechanism independent of TMPRSS2, with these characteristics corresponding to distinct regions of the spike protein. The receptor-binding domain was responsible for impaired cell fusion, while the S2 domain was important for endosomal fusion. The fast global spread and increased virulence of the Omicron variant may be attributable to changes in vaccines and antigenicity^35^. Studies have indicated that the *R*_*0*_ of the Omicron variant reaches 8.2, and the transmission ability is exceedingly high, which is greater than the assumed transmissibility in our simulation^36^. Therefore, we re-simulated according to the control strategies of the Guangzhou epidemic. If the same measures are followed, the epidemic size stimulated by our model will be reached 695 cases (*R*_*0*_: 8.2, incubation: 3 days), indicating that more stringent measures than strategy 1 (such as quarantine and social distancing measures are implemented in advance) are needed in order to contain the outbreak. On April 8, 2022, the Omicron variant was introduced in Guangzhou. Due to the understanding of the high transmissibility of the Omicron variant, Guangzhou government implemented and strengthen the same measures against Delta in 2021 (Strategy 1). Since April 8 nucleic acid screening testing and close contacts were immediately investigated. It is worth noting that, routine surveillance on the high risk population has been set on since the Delta variant outbreak. In addition, Baiyun District (the epicenter of the epidemic) was separated into three zones: lockdown zone, controlled zone, and precautionary zone. On April 9, a series of social distancing measures (shops, Internet cafés, amusement places, etc. were closed, schools switched to online education, etc.). Overall, social distancing measures have been adopted even earlier than that in the Delta epidemic in 2021^37,38^. As a consequence, *R*_*t*_ decreased to 1 on April 20, which demonstrates that the control strategies were still successful. Although the Omicron variant has a low case fatality rate compared to other VOCs, it is highly transmissible and may quickly break the local medical defensive line, resulting in a considerable rise in the number of cases and fatalities^39^. Significantly minimize the number of infections and guarantee that small children and the elderly will not be infected with Omicron and cause a significant number of fatalities^40^. In the face of future variants, it is prudent to pay special attention to their transmissibility, immune escape and clinical symptoms, and the performance of control measures also has to be changed accordingly.

Our study has several limitations. First, we focused mainly on the impact of control strategies to reduce transmission, but have not evaluated the economic and social costs associated with these measures. Second, we have not assessed the impact of personal prevention measures such as wearing masks, which were difficult to quantify and measure. Third, the optimal timing and duration for implementing each intervention were not analyzed.

To our best knowledge, this is the first study to model and assess containment strategies for the Delta variant. There are different control policies across the world, but our findings would be suggestive to other countries.

## Conclusions

In summary, we analyzed the control strategies which were able to contain a sizeable outbreak of the Delta variant in a community setting. Social distancing measures were found to be a critical component, while early quarantine/isolation were also helpful to slow down disease spread. We also considered alternative control strategies which may benefit countries or regions which attempt to contain a localized outbreak of the Delta variant.

## Supplementary Information

Supplementary Information.

## Data Availability

The datasets used and/or analysed during the current study available from the corresponding author on reasonable request.

## Abbreviations

SARS-CoV-2: Severe acute respiratory syndrome coronavirus 2
COVID-19: Coronavirus disease 2019
WHO: World Health Organization
VOC: Variants of concern
NGS: Next generation sequencing
NPIs: Non-pharmaceutical interventions
CDC: Center for Disease Control and Prevention
IQR: Interquartile range
CI: Confidence interval
