# Supplementary material for: Containment of SARS-CoV-2 Delta strain in Guangzhou, China by quarantine and social distancing: a modelling study
Source: Sci Rep. 2022 Dec 6;12:21096. doi: 10.1038/s41598-022-21674-7 (PMC9727161; doi:10.1038/s41598-022-21674-7)
Supplement: Supplementary file 1 — Supplementary Information. [file 41598_2022_21674_MOESM1_ESM.docx]

**Supplementary Information**

[**Sensitivity**](javascript:;) [**analysis**](javascript:;)**
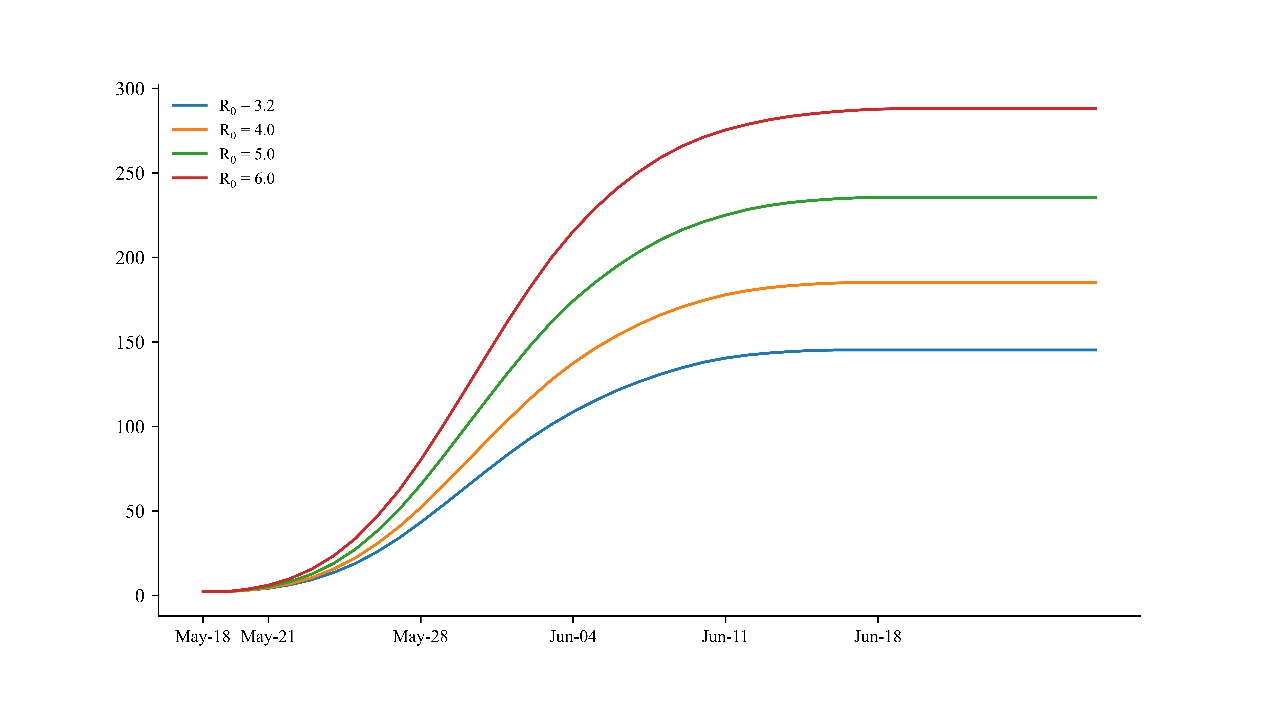
**

**Figure S1. Sensitivity of total infections to the change basic of reproduction number (**$\mathbf{R}_{\mathbf{0}}$**)**

**
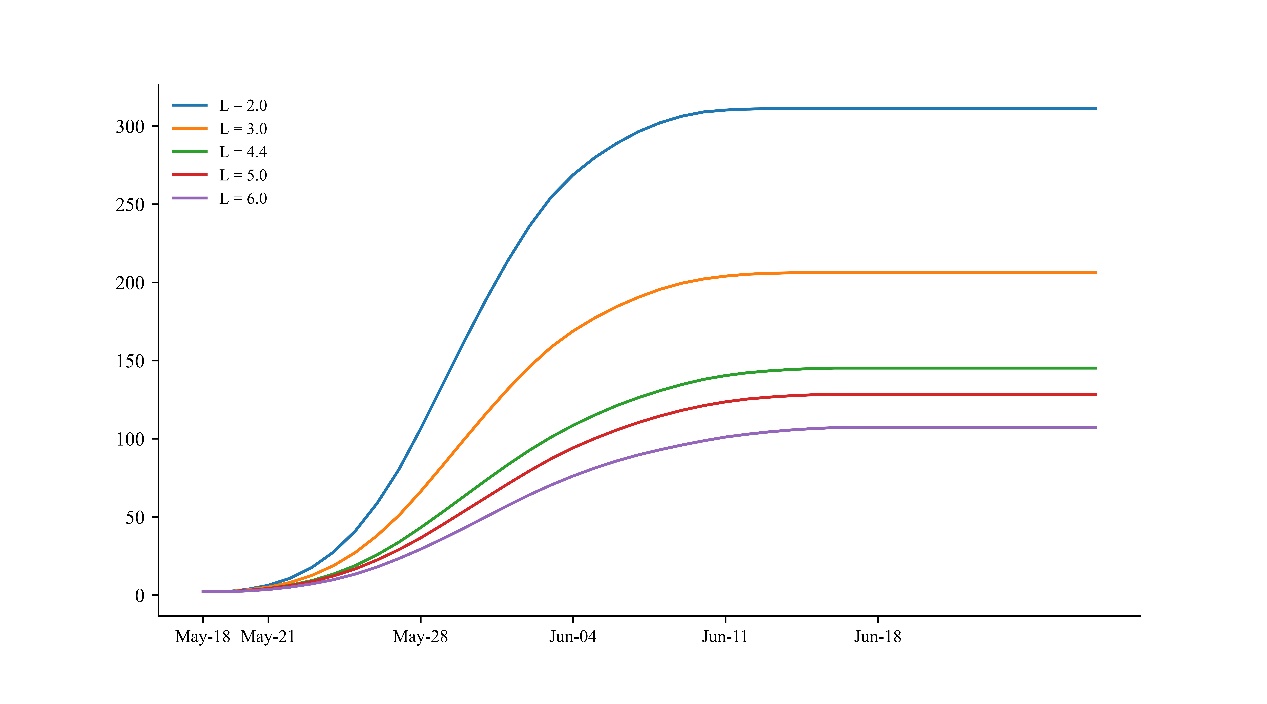
**

**Figure S2. Sensitivity of total infections to the change of incubation period (*L*)**

**
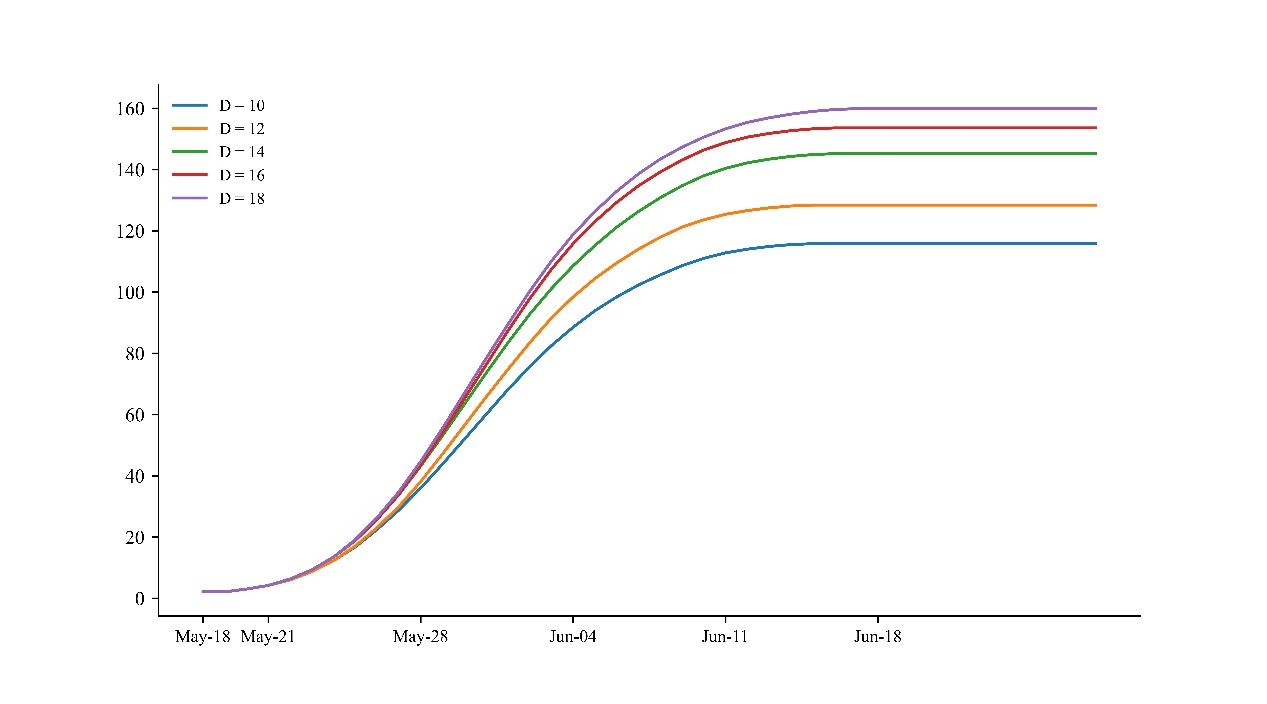
**

**Figure S3. Sensitivity of total infections to the change of recovery period (*D*)**


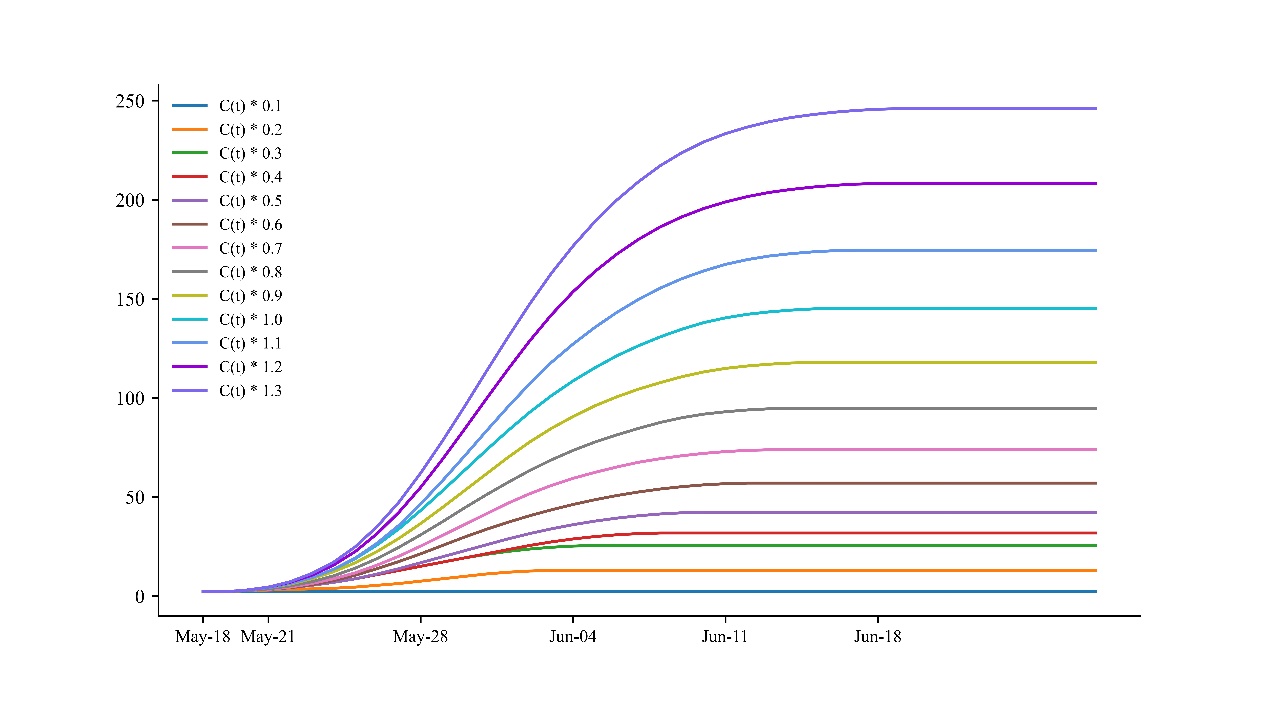


**Figure S4. Sensitivity of total infections to the change of close contact rate** $\boldsymbol{C(t)}$


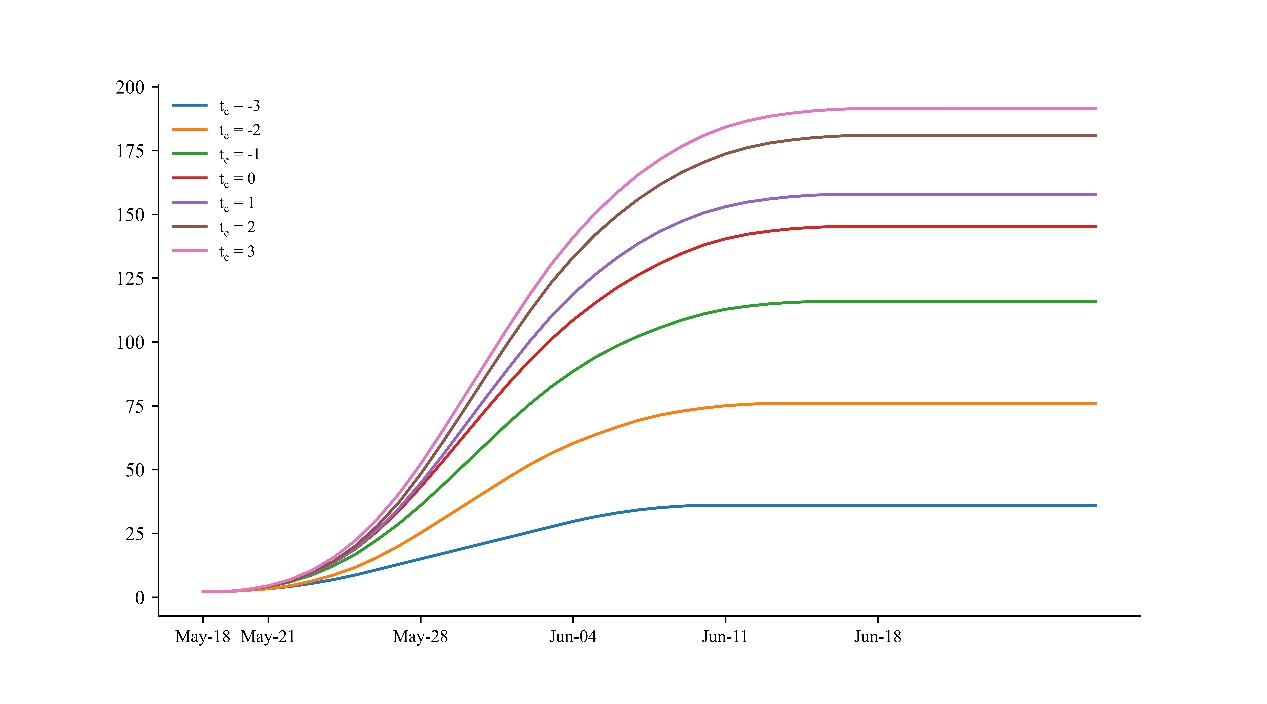


**Figure S5. Sensitivity of total infections to the change of time from onset of infectiousness to quarantine/isolation (**$\boldsymbol{t}_{\boldsymbol{c}}$**)**


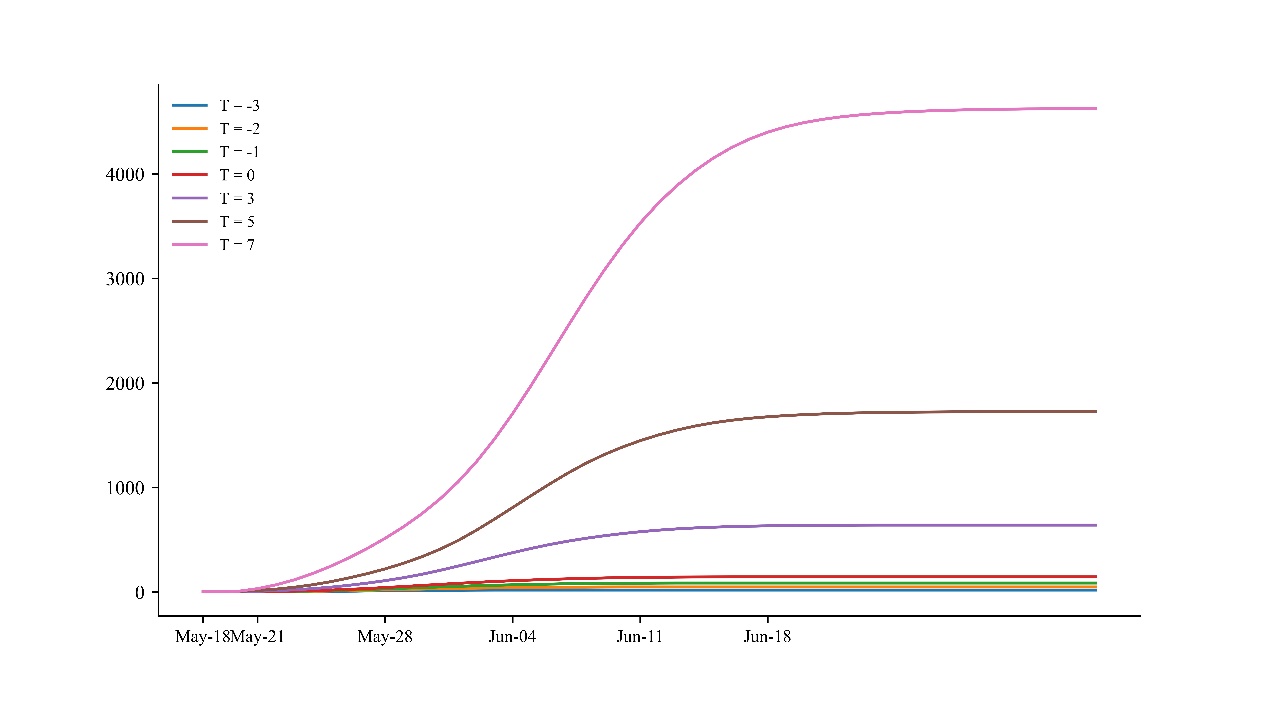


**Figure S6. Sensitivity of total infections to the change of time of control measurements (**$\boldsymbol{T}$**)**

**Table S1. Average contact rate and number of close contacts in the model**

|  | Pre-phase | The phase I | The phase II | The phase III | The phase IV |
| --- | --- | --- | --- | --- | --- |
| The average contact rate | 31 | 29 | 7 | 7 | 0 |
| The total number of close contacts | 305 | 4905 | 4759 | 1426 | 0 |

**Table S2. Time from symptom onset to control (quarantine/isolation)**

| **Case** | **Onset** | **Control** | **Days** |
| --- | --- | --- | --- |
| **1** | May-18 | May-21 | 3 |
| **2** | May-24 | May-21 | -3 |
| **3** | May-25 | May-26 | 1 |
| **4** | May-24 | May-26 | 2 |
| **5** | May-26 | May-26 | 0 |
| **6** | May-27 | May-26 | -1 |
| **7** | Jun-03 | May-26 | -8 |
| **8** | May-26 | May-26 | 0 |
| **9** | May-26 | May-26 | 0 |
| **10** | May-27 | May-26 | -1 |
| **11** | May-27 | May-26 | -1 |
| **12** | May-31 | May-27 | -4 |
| **13** | May-27 | May-26 | -1 |
| **14** | May-27 | May-26 | -1 |
| **15** | May-28 | May-26 | -2 |
| **16** | May-29 | May-28 | -1 |
| **17** | Jun-01 | May-29 | -3 |
| **18** | Jun-01 | May-28 | -4 |
| **19** | May-28 | May-26 | -2 |
| **20** | May-29 | May-29 | 0 |
| **21** | May-29 | May-29 | 0 |
| **22** | May-29 | May-29 | 0 |
| **23** | May-29 | May-28 | -1 |
| **24** | Jun-02 | May-29 | -4 |
| **25** | May-29 | May-29 | 0 |
| **26** | Jun-03 | May-29 | -5 |
| **27** | May-29 | May-30 | 1 |
| **28** | May-28 | May-28 | 0 |
| **29** | May-27 | May-30 | 3 |
| **30** | May-29 | May-28 | -1 |
| **31** | May-27 | May-30 | 3 |
| **32** | May-29 | May-30 | 1 |
| **33** | May-30 | May-30 | 0 |
| **34** | May-30 | May-30 | 0 |
| **35** | May-31 | May-31 | 0 |
| **36** | May-28 | May-31 | 3 |
| **37** | May-31 | May-31 | 0 |
| **38** | May-29 | May-31 | 2 |
| **39** | May-29 | May-31 | 2 |
| **40** | Jun-02 | May-31 | -2 |
| **41** | May-31 | May-31 | 0 |
| **42** | May-30 | May-26 | -4 |
| **43** | May-31 | May-31 | 0 |
| **44** | Jun-04 | May-31 | -4 |
| **45** | Jun-02 | May-28 | -5 |
| **46** | May-31 | May-31 | 0 |
| **47** | May-29 | Jun-01 | 3 |
| **48** | May-31 | May-29 | -2 |
| **49** | May-27 | Jun-01 | 5 |
| **50** | Jun-05 | Jun-01 | -4 |
| **51** | May-24 | Jun-01 | 8 |
| **52** | Jun-01 | Jun-01 | 0 |
| **53** | Jun-01 | May-29 | -3 |
| **54** | Jun-03 | May-31 | -3 |
| **55** | May-29 | May-30 | 1 |
| **56** | Jun-01 | May-30 | -2 |
| **57** | Jun-01 | May-30 | -2 |
| **58** | May-31 | Jun-01 | 1 |
| **59** | May-29 | May-30 | 1 |
| **60** | Jun-05 | Jun-01 | -4 |
| **61** | May-31 | May-30 | -1 |
| **62** | Jun-01 | Jun-01 | 0 |
| **63** | Jun-01 | May-30 | -2 |
| **64** | Jun-01 | May-30 | -2 |
| **65** | Jun-01 | May-30 | -2 |
| **66** | May-31 | May-30 | -1 |
| **67** | May-31 | May-30 | -1 |
| **68** | May-26 | Jun-02 | 7 |
| **69** | Jun-01 | Jun-02 | 1 |
| **70** | Jun-02 | Jun-02 | 0 |
| **71** | May-31 | May-28 | -3 |
| **72** | Jun-01 | Jun-01 | 0 |
| **73** | May-31 | Jun-01 | 1 |
| **74** | Jun-02 | May-30 | -3 |
| **75** | Jun-02 | Jun-01 | -1 |
| **76** | Jun-03 | Jun-02 | -1 |
| **77** | Jun-02 | Jun-03 | 1 |
| **78** | Jun-03 | Jun-03 | 0 |
| **79** | May-30 | May-26 | -4 |
| **80** | Jun-01 | May-26 | -6 |
| **81** | Jun-03 | Jun-03 | 0 |
| **82** | Jun-08 | Jun-02 | -6 |
| **83** | Jun-04 | Jun-02 | -2 |
| **84** | Jun-05 | Jun-03 | -2 |
| **85** | Jun-05 | Jun-03 | -2 |
| **86** | Jun-05 | Jun-03 | -2 |
| **87** | Jun-05 | Jun-04 | -1 |
| **88** | Jun-06 | May-29 | -8 |
| **89** | Jun-04 | Jun-06 | 2 |
| **90** | Jun-02 | Jun-06 | 4 |
| **91** | Jun-05 | Jun-06 | 1 |
| **92** | Jun-05 | Jun-06 | 1 |
| **93** | Jun-06 | Jun-05 | -1 |
| **94** | May-28 | Jun-06 | 9 |
| **95** | Jun-01 | Jun-05 | 4 |
| **96** | Jun-05 | Jun-07 | 2 |
| **97** | Jun-05 | Jun-07 | 2 |
| **98** | Jun-03 | Jun-07 | 4 |
| **99** | Jun-02 | Jun-07 | 5 |
| **100** | Jun-03 | Jun-07 | 4 |
| **101** | Jun-07 | Jun-05 | -2 |
| **102** | Jun-06 | Jun-05 | -1 |
| **103** | Jun-06 | Jun-05 | -1 |
| **104** | Jun-04 | Jun-05 | 1 |
| **105** | Jun-07 | Jun-05 | -2 |
| **106** | Jun-05 | Jun-03 | -2 |
| **107** | Jun-07 | Jun-06 | -1 |
| **108** | Jun-07 | Jun-05 | -2 |
| **109** | Jun-07 | Jun-07 | 0 |
| **110** | Jun-08 | May-29 | -10 |
| **111** | Jun-05 | May-29 | -7 |
| **112** | Jun-06 | Jun-07 | 1 |
| **113** | Jun-03 | Jun-07 | 4 |
| **114** | Jun-08 | Jun-07 | -1 |
| **115** | Jun-08 | Jun-07 | -1 |
| **116** | May-31 | Jun-07 | 7 |
| **117** | Jun-09 | Jun-03 | -6 |
| **118** | Jun-09 | Jun-09 | 0 |
| **119** | Jun-10 | Jun-10 | 0 |
| **120** | Jun-10 | Jun-10 | 0 |
| **121** | Jun-07 | Jun-07 | 0 |
| **122** | Jun-08 | Jun-05 | -3 |
| **123** | Jun-10 | Jun-07 | -3 |
| **124** | Jun-10 | Jun-07 | -3 |
| **125** | Jun-06 | Jun-07 | 1 |
| **126** | Jun-04 | Jun-07 | 3 |
| **127** | Jun-08 | Jun-09 | 1 |
| **128** | Jun-09 | Jun-05 | -4 |
| **129** | Jun-11 | Jun-11 | 0 |
| **130** | Jun-10 | Jun-10 | 0 |
| **131** | Jun-10 | Jun-07 | -3 |
| **132** | Jun-07 | Jun-12 | 5 |
| **133** | Jun-03 | Jun-07 | 4 |
| **134** | Jun-11 | Jun-07 | -4 |
| **135** | Jun-13 | Jun-07 | -6 |
| **136** | Jun-13 | Jun-09 | -4 |
| **137** | Jun-13 | Jun-08 | -5 |
| **138** | Jun-12 | Jun-07 | -5 |
| **139** | Jun-12 | Jun-07 | -5 |
| **140** | Jun-16 | Jun-07 | -9 |
| **141** | Jun-15 | Jun-07 | -8 |
| **142** | Jun-12 | Jun-07 | -5 |
| **143** | Jun-15 | Jun-07 | -8 |
| **144** | Jun-18 | Jun-07 | -11 |
| **145** | Jun-18 | Jun-07 | -11 |
| **146**  **147**  **148**  **149**  **150**  **151**  **152**  **153** | Jun-3  Jun-3  Jun-9  Jun-1  May-29  Jun-2  Jun-3  Jun-3 | -  -  -  -  -  -  -  - | -  -  -  -  -  -  -  - |

“-” means no data
